# Behavioral and oscillatory signatures of switch costs in highly proficient bilinguals

**DOI:** 10.1038/s41598-023-34895-1

**Published:** 2023-05-12

**Authors:** Polina Timofeeva, Ileana Quiñones, Shuang Geng, Angela de Bruin, Manuel Carreiras, Lucia Amoruso

**Affiliations:** 1 BCBL, Basque Center On Brain, Language and Cognition, Paseo Mikeletegi 69, 2nd floor, 20009 Donostia/San Sebastian, Spain; 2 Universidad del País Vasco (UPV/EHU), 20009 San Sebastian, Spain; 3 Department of Psychology, University of York, York, YO10 5DD UK; 4 Ikerbasque, Basque Foundation for Science, 48940 Bilbao, Spain

**Keywords:** Neuroscience, Cognitive neuroscience, Language

## Abstract

Bilinguals with a high proficiency in their first (L1) and second language (L2) often show comparable reaction times when switching from their L1 to L2 and vice-versa (“symmetrical switch costs”). However, the neurophysiological signatures supporting this effect are not well understood. Here, we ran two separate experiments and assessed behavioral and MEG responses in highly proficient Spanish-Basque bilinguals while they overtly name pictures in a mixed-language context. In the behavioral experiment, bilinguals were slower when naming items in switch relative to non-switch trials, and this switch cost was comparable for both languages (symmetrical). The MEG experiment mimicked the behavioral one, with switch trials showing more desynchronization than non-switch trials across languages (symmetric neural cost) in the alpha band (8–13 Hz). Source-localization revealed the engagement of right parietal and premotor areas, which have been linked to language selection and inhibitory control; and of the left anterior temporal lobe (ATL), a cross-linguistic region housing conceptual knowledge that generalizes across languages. Our results suggest that highly proficient bilinguals implement a language-independent mechanism, supported by alpha oscillations, which is involved in cue-based language selection and facilitates conceptually-driven lexical access in the ATL, possibly by inhibiting non-target lexical items or disinhibiting target ones.

## Introduction

Nowadays, bilingualism – mastery of more than one language^1^ – is not an exception but common practice in many societies. Some countries estimate the percentage of bilinguals to be above 65% and constantly growing [EuroStat, 2016]. In daily life, bilingual speakers seem to fluently switch from one language to another while avoiding cross-language interference. However, to achieve this apparent effortless behavior, bilinguals need to control their languages in use. Most of the experimental evidence from bilingual language control comes from studies using language-switching paradigms in which participants have to alternate between two languages in response to cues. For example, a bilingual might be instructed to name pictures in Language A when the picture is presented with a red cue but use Language B when the cue is blue. This includes switch trials (requiring alternations between the two languages) and non-switch trials (where participants stay in the same language). Typically, higher error rates and longer naming latencies are observed on the switch compared to non-switch trials, with the difference between them referred to as the switch cost effect (e.g.^2^).

Unbalanced bilinguals, who are more proficient in their dominant first language (L1) than in their weaker second language (L2), typically show larger switch costs when switching from the L2 to the L1 than vice versa^2^. One of the most prominent models of bilingual language control, the Inhibitory Control Model^3,4^, explains this paradoxical asymmetry via an inhibitory mechanism, in which the magnitude of the suppression is proportional to language proficiency and activation. Given that a more proficient language (L1) might be more active, more inhibition would be applied to suppress L1 interference during L2 naming and, hence, more effort/time would be required to release its residual effect when switching back to the L1. In other words, if there is a difference in proficiency between the two languages, the switch cost should be asymmetric because more inhibition is required to suppress the dominant L1.

On the other hand, this model predicts that for bilinguals showing similar proficiency in both languages, the switch cost should be symmetric, given that the amount of inhibition deployed to control for activation across languages should be equivalent. This prediction is indeed supported by behavioral data from highly proficient bilinguals showing symmetric switch costs when alternating between languages during picture-naming tasks in mixed-language contexts^5–10^.

From a neuroanatomical standpoint, numerous neuroimaging studies have explored the neural substrates supporting language switching in bilinguals. These studies have pointed to the involvement of cortical and subcortical regions related to language control but also to domain-general executive control^11^. Among these regions, the most commonly reported are the caudate, the anterior cingulate cortex (ACC), the supplementary motor area (SMA), the prefrontal cortex (PFC), the inferior frontal gyrus (IFG), and temporo-parietal areas comprising the supramarginal gyrus (SMG) and the superior frontal gyrus (SFG)^3,8,9,12–15^.

When considering the timing of switching in bilinguals, previous neurophysiological evidence from M/EEG studies focused on evoked responses (ERPs/ERFs) indicates that language control effects (i.e. switch vs. non-switch trials) emerge between ~ 200 ms and 600 ms after stimulus onset. This time window corresponds to the classical N2 and N400 components^16,17^. On the one hand, early N2 modulations have been proposed to reflect top-down control, possibly in the form of response inhibition or disengagement from the non-target language, with larger N2 responses for the switch as compared to non-switch trials, and with this effect being present only in the less dominant language^18–20^. On the other hand, N400 responses occurring later in time are thought to index semantic aspects of language processing. N400 modulations following switch trials have been suggested to reflect a greater demand in the integration of lexical and semantic representations^21^ or the effort necessary to overcome the inhibition of the previous language^22^. Earlier studies indicate that this component is sensitive to language proficiency, with individuals with low proficiency in their L2 showing deviant N400 responses^23,24^. Interestingly, recent evidence^25^ from balanced, highly proficient bilinguals shows similar N400 semantic responses across L1 and L2, suggesting that, as language proficiency improves, conceptual representations become semantically processed in the same way in the L1 and the L2^3,26^.

However, classical time-locked ERP/ERF analysis is blind to information not phase-locked to the stimuli, resulting in less sensitivity when tapping into ongoing neurocognitive dynamics associated with bilingual language processing^27,28^. Relative to ERP/ERF, time–frequency analysis can better characterize the temporal dynamics of oscillations contained in the brain signal. Indeed, oscillatory activity is thought to play a key role in neural communication and to reflect distinct cognitive operations at different frequency bands^29^, providing a fine-grained characterization of neurophysiological mechanisms supporting cognition. Previous findings point to the involvement of two main oscillations in bilingual language control: theta (4–7 Hz) and alpha (8–13 Hz) rhythms^17^. On the one hand, theta power increases have been reported for L2 as compared to L1 switching during speech production in low proficient Chinese-English bilinguals with high inhibitory control abilities, possibly indexing cross-language interference at the lexical selection level^30^. Another study on word production in unbalanced Dutch-English bilinguals^31^, found theta power increases after participants selected the wrong language for speaking during cued language switching, reflecting a role for theta in the monitoring of speech errors. Similarly, in non-linguistic tasks in which participants have to deal with conflicting information (e.g. Go-no go, Flanker task), theta power increases have been observed in incongruous as compared to congruous trials^32–34^, thus supporting its broader involvement in executive control (e.g. conflict monitoring) under situations of increased cognitive demands.

On the other hand, oscillatory activity in the alpha frequency band (8–13 Hz) has been consistently linked to functional inhibition^35–37^. Under this view, alpha is considered a general mechanism that subserves various cognitive processes that use inhibitory control in tasks requiring interference suppression. When considering language control, bilinguals tend to exhibit overall higher alpha power than monolinguals^38^, with this power correlating with L1 and L2 experience-related measures. Furthermore, alpha oscillations have been linked to lexico-semantic access in highly proficient bilinguals^39^ and are thought to shape inhibition in semantic association networks, allowing the controlled retrieval of information from long-term memory^36,40^.

Altogether, these findings suggest that theta and alpha frequency bands might play a key role in bilingual language control. Nevertheless, only a few studies have attempted to investigate the oscillatory dynamics subserving this process during speech production^30^. Furthermore, to the best of our knowledge, no study has approached the topic while considering highly proficient bilinguals. To address this gap, we separately assessed behavioral and MEG responses in two independent groups of highly proficient Spanish-Basque bilinguals while they performed a picture-naming task requiring the utterance of nouns in a mixed-language context (i.e. alternating between languages within the same block depending on a color cue).

In line with previous evidence suggesting that highly proficient bilinguals show similar behavioral switch costs when switching from L1 to L2 and from L2 to L1, we expected symmetrical switch costs for Spanish and Basque. At the oscillatory level, we predicted similar differences between switch and non-switch trials across languages (i.e. symmetric neural costs) likely reflected in theta and alpha modulations between ~ 200 ms and ~ 600 ms, with this effect potentially engaging language control and more domain-general cognitive control regions.

## Results

### Online behavioral results

**Figure 1:**
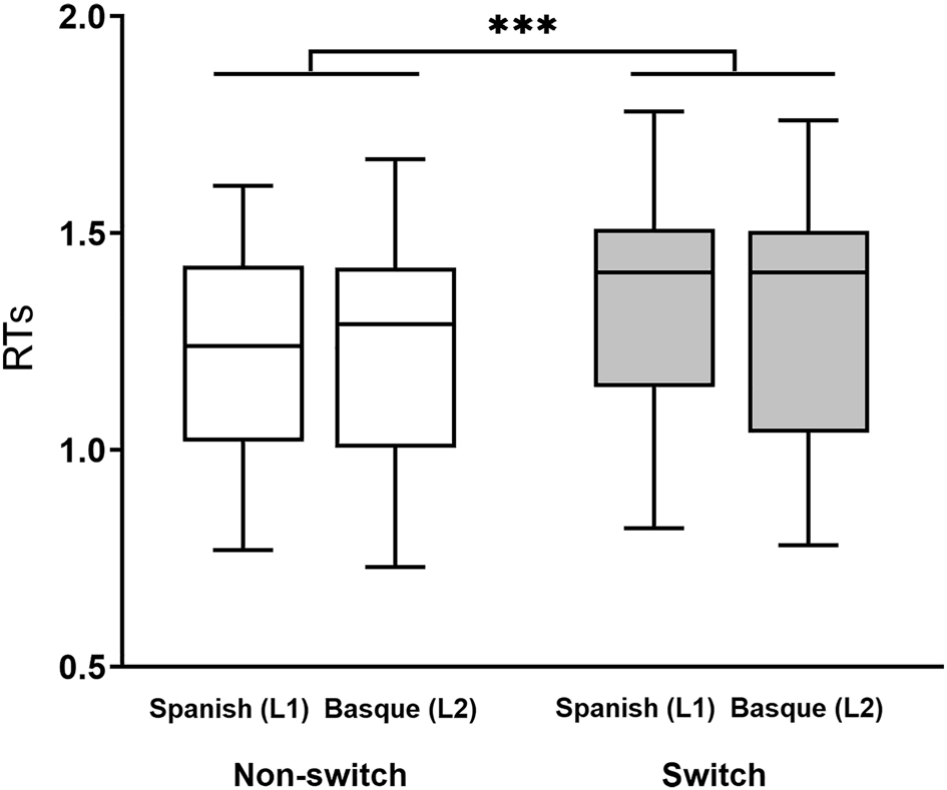
Online behavioural results. Mean reaction times (in seconds) for non-switch and switch trials in Spanish (L1) and Basque (L2). Asterisks indicate significant difference between switch and non-switch conditions (***p < 0.001) obtained in the two-way ANOVA. Bars indicate SD.

The 2-way ANOVA performed on the RTs obtained in the online experiment showed a main effect of Trial type (*F*_1, 20_ = 35.10, *p* < 0.0001, *η*_p_^2^ = 0.64), suggesting that switch trials (Mean = 1.33 secs; SD = 0.251 secs) were named more slowly than non-switch trials (Mean = 1.24 secs; SD = 0.236 secs). No main effect of Language (*F*_1, 20_ = 3.18, *p* = 0.09, *η*_p_^2^ = 0.14) nor the interaction between Trial type and Language (*F*_1, 20_ = 2.34, *p* = 0.14, *η*_p_^2^ = 0.11) reached significance, suggesting that switch costs across Spanish (Mean = 0.106 secs) and Basque (Mean = 0.073 secs) were similar. See Fig. 1.

Because the lack of a significant interaction does not necessarily mean evidence for the absence of an effect, we conducted a Bayesian ANOVA that allows quantifying evidence for and against its presence. We used the JASP software^41^ with its default prior (*Cauchy* distribution, r = 0.5), and computed the inclusion Bayes Factor (BF_incl_) for each main effect and interaction. Briefly, BF_incl_ indicates how much more likely the data are under models that include a particular predictor compared with those that do not, allowing to determine the relative strength of evidence for each factor on the dependent variable. A BF_incl_ > 1 provides evidence for inclusion, whereas a BF_incl_ < 1, indicates evidence for exclusion^42^.

Our analysis revealed overwhelming evidence in favor of the inclusion of the Trial type factor (BF_incl_ = 3.996e + 6), indicating that models that include this factor are 3.996e + 6 times more likely than those that do not. However, we found evidence against inclusion for both Language (BF_incl_ = 0.66) and the interaction between Trial type and Language (BF_incl_ = 0.57). Overall, this supports the conclusion of the frequentist ANOVA showing that switching effects were similar across languages.

Finally, we ran a third analysis using linear mixed-effects models (LMMs) to model individual variation in the data. In this analysis, we included Language, Trial type, and their interaction as fixed effects and participants and items (images) as random effects. Overall, results from the LMMs were comparable to those obtained with the ANOVA. While the effect of Trial type was significant (*F* = 6.16, *p* = 0.03), the effect of Language (*F* = 0.319, *p* = 0.58) and the interaction between Language and Trial type did not reach significance (*F* = 0.003, *p* = 0.95).

### Sensor level results

**Figure 2:**
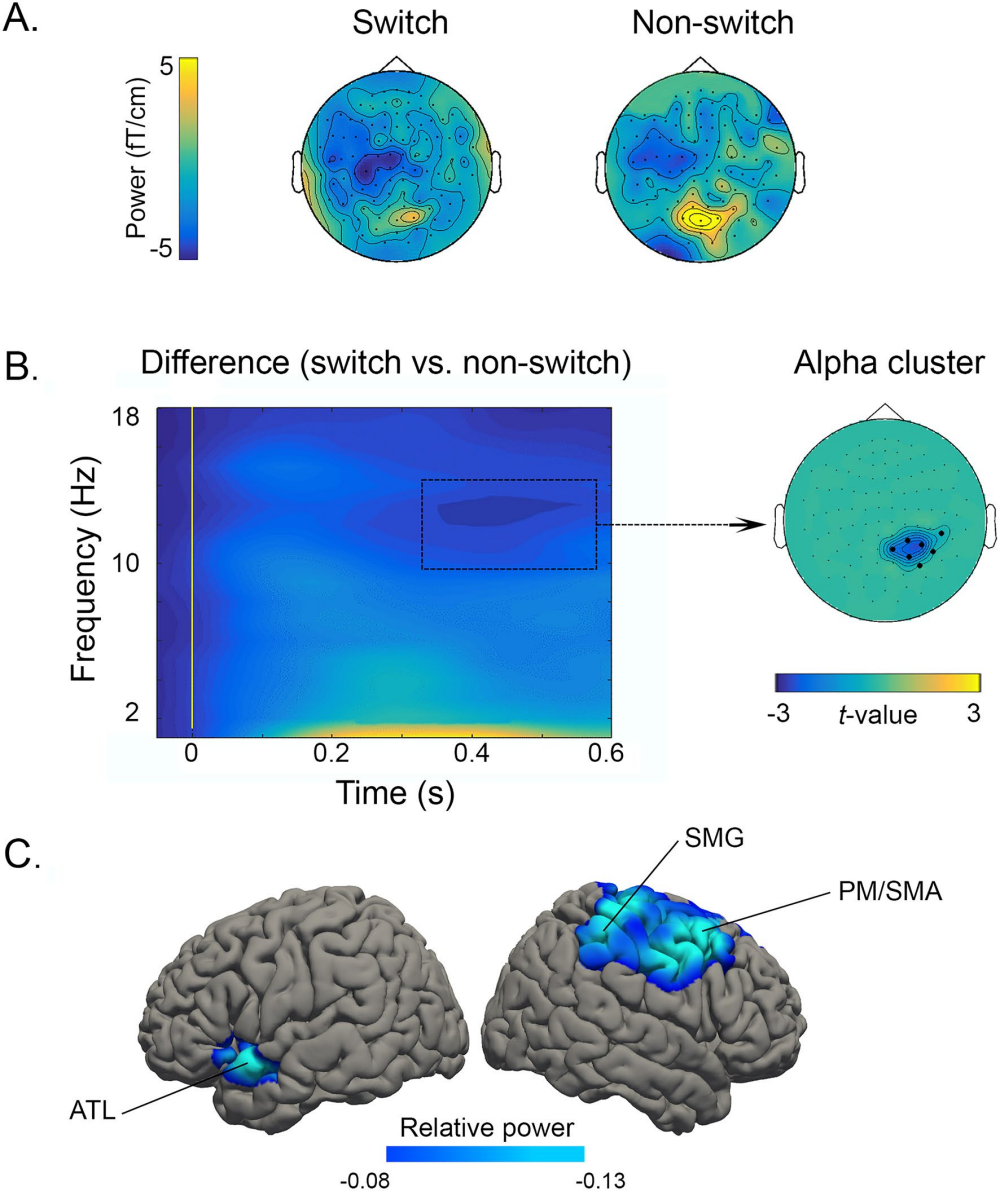
Oscillatory effects in bilingual speakers. (**A**) Topographic distribution plots for switch and non-switch conditions in the alpha frequency band (8–13 Hz) between 350 and 600 ms after object picture onset. (**B**) Time–frequency representation (TFR) showing the difference between switch and non-switch conditions across languages in the combined gradiometers highlighted by the significant alpha negative cluster. (**C**) Localization of alpha peaks (switch vs. non-switch) circumscribed to the time interval highlighted by the significant cluster. All plotted regions reached a *p*-value < 0.01.

To analyze responses obtained in the MEG experiment, we used a cluster-based permutation approach to test for an effect of Language (Spanish vs. Basque) and Trial type (switch vs. non-switch). Then, following state-of-the-art pipelines for testing an interaction effect via means of a cluster-based permutation approach (www.fieldtriptoolbox.org/faq/how_can_i_test_an_interaction_effect_using_cluster-based_permutation_tests/); we subtracted switch and non-switch conditions within each language and compared the two differences. Overall, the effect of Language did not reach significance (all *p*s > 0.05) in any of the frequency bands (i.e. theta and alpha) or time windows (i.e. early and late). However, there was a significant effect of Trial type (Fig. 2A), as highlighted by a negative cluster in the alpha frequency band (8–13 Hz; Monte Carlo *p* = 0.01, two-tailed), showing stronger power decreases for the switch condition as compared to the non-switch one. This effect occurred in the late time window (i.e. 350-600 ms) and was localized in the right combined gradiometers (Fig. 2B). No Trial type effects were observed in the theta band. Finally, the Language x Trial type interaction did not reach significance (all *p*s > 0.05) in any of the frequency bands (i.e., theta and alpha) or time windows (i.e. early and late).

### Source localization of the MEG sensor-level results

Oscillatory effects at the sensor level were reconstructed at the source level on the frequency band and time window highlighted by the significant cluster (i.e. 8–13 Hz between 350 and 600 ms). Alpha peaks identified for the switching effect (switch vs. non-switch trials across languages) were localized in the right supramarginal gyrus (BA40), the right premotor/supplementary motor area (BA6), and the left anterior temporal lobe (BA38, see Fig. 2C).

## Discussion

In the present study, we aimed to investigate the behavioral and oscillatory signatures supporting language control in highly proficient bilinguals by examining language switching during speech production. To this end, we ran two separate experiments in independent samples of highly proficient Spanish-Basque bilinguals and acquired behavioral and MEG responses during an overt picture naming task in a mixed-language context. Results from the behavioral experiment, revealed overall slower responses in switch as compared to non-switch trials (i.e. a switch cost). Importantly, this switch cost was comparable across the two languages, replicating previous findings of symmetrical switch effects in bilinguals with similar L1-L2 proficiency. Time–frequency results from the MEG experiment, revealed comparable neural switch costs in Spanish and Basque, showing significant power decreases in the alpha frequency band (8–13 Hz) between 350 and 600 ms after picture onset for switch compared to non-switch trials, irrespectively of the language at use. This effect was source-localized in domain-general (e.g. right SMG and PM/SMA) and language-specific (left ATL) control regions. Overall, we report behavioral and neuromagnetic evidence on the existence of a common (i.e., same for the two languages) control mechanism in highly proficient bilinguals, which supports cue-based language selection and controlled access to lexico-semantic representations during speech production.

When considering behavioral findings, bilingual speakers exhibited language switch costs (i.e. increased reaction times for switch vs. non-switch trials). This effect is well predicted by previous literature^43^, suggesting that switching involves an effort associated with system reconfiguration (i.e. choosing a different language from the one previously used). According to the IC model, one of the key mechanisms that underpin language switching is inhibition: bilingual speakers suppress the non-target language to properly produce a response in the target one^3,4,44^. According to this view, if L1 and L2 proficiency levels are balanced, symmetric switch costs should be observed, indicating that the amount of inhibition deployed to control for language interference is similar. Our findings of symmetric switch costs in Spanish and Basque align well with the IC model and with previous behavioral evidence on cued language switching^5–10,45^, suggesting the existence of comparable levels of control when bilinguals are highly proficient in both languages and acquire both during early childhood.

A similar pattern of responses was observed when considering MEG results, with bilinguals showing equivalent neural switch costs across languages (i.e. no effect of language nor interaction between language and trial type). Switch and non-switch conditions (collapsed across languages) significantly differed between 350 and 600 ms after picture onset, with alpha power (8–13 Hz) decreases being stronger for the switch compared to non-switch trials. From a broader standpoint, alpha oscillations have been proposed as a hallmark of inhibitory control and, in particular, as a fingerprint of controlled access to semantic knowledge stored in long-term memory^36,37,46^. In the language domain, alpha power decreases between ~ 300 ms and 500 ms after picture onset have been linked to the lexico-semantic processing of object-related knowledge during speech production in monolingual^31,47^ individuals. Furthermore, a similar spectro-temporal pattern has also been found in highly proficient bilinguals when naming pictures either in their L1 or L2^39^. This aligns well with proposals suggesting that as language proficiency improves, L1 and L2 representations become semantically processed similarly^3,26^.

In the context of language switching, while there is evidence for a role of alpha power decreases in language control during comprehension^48^, to the best of our knowledge, this is the first study reporting its involvement during overt speech production.

Source localization of the alpha effect highlighted the involvement of language-specific (i.e. left ATL) and more domain-general executive control regions (i.e. right SMG and PMC/SMA). In highly proficient bilinguals, a similar activation pattern showing greater activity in the right SMG (BA40) and PMC/SMA regions (BA6) has been recently reported in neuroimaging studies for switch as compared to non-switch trials^8,9,49^. On the one hand, it has been proposed that bilateral inferior parietal cortices mediate language selection^50^. In particular, during switching, the right SMG's role would be biasing selection towards the target language – while its left counterpart would be responsible for biasing selection away from the language no longer in use. Interestingly, a recent study^51^ applying anodal transcranial direct current stimulation (tDCS) over the right parietal cortex shows a selective improvement in performance when switching to a recently inhibited task, supporting a broader role of this region in overcoming previous inhibition. Similarly, the SMA region has been implicated in proactive switching^52^, namely, in using contextual sensory cues hinting at the need for a change towards a new behavior. For instance, disruptive repetitive transcranial magnetic stimulation over the SMA selectively hampers individuals’ performance during the cue period that signals a switch trial^53^, suggesting that this region mediates cue-based prospective reconfiguration. This proposal aligns well with the cued language switching paradigm used in the present study, in which a color cue indicated to bilinguals whether a language change was required or not. Thus, the finding of parieto-prefrontal alpha oscillations mediating switching may reflect target language selection based on cue processing.

On the other hand, the left ATL (BA38) is proposed to play a key role in mapping concepts to words during speech production, with damage to this area resulting in specific deficits during conceptually-driven word retrieval^54,55^. Importantly, the ATL is thought to house language-invariant semantic representations in bilinguals^39,56–58^ and, in particular, object-related ones^59–61^. Indeed, it has been recently shown that highly proficient bilinguals recruit the ATL during object picture naming^39^. This finding aligns well with evidence suggesting that ATL involvement is critical when accessing conceptual knowledge from visual inputs^62^. Furthermore, the superior part of the left ATL exhibits strong connectivity with other areas highlighted by our source analysis (i.e. SMG and PM/SMA) during both task and rest^63^ and with the right parietal cortex during language switching in highly proficient bilinguals^56^. Of note, it has been shown^64^ that left ATL contribution to semantic processing is critical around 400 ms after stimulus onset, a time window that aligns well with our findings. Taken together, the source level results of the alpha effect suggest that the right parieto-prefrontal network may be involved in biasing language selection based on cue information. This network would facilitate conceptually-driven lexical access in the ATL, possibly by inhibiting non-target lexical items and/or disinhibiting target ones.

### Limitations and avenues for further research

The present study is not without limitations. First, it is worth noting that, in our cued language switching paradigm, we used only one color cue per language. This leads to a potential confound between cue switching and language switching, which may explain our switch costs results (i.e. a language switch is also by definition a cue switch while a language non-switch is also by definition a cue non-switch). Nevertheless, based on previous studies^10,65^, we find this possibility unlikely. Indeed, it has been shown that even when cue-switch and language-switch are dissociated (e.g. by using multiple color cues per language), language-related switch costs are still substantial. Notably, one of these studies^10^ tested an equivalent group of highly proficient Spanish-Basque bilinguals and reported symmetrical switching costs similar to those found here, indicating that comparable results can be achieved when the cue and language switch are not confounded.

Second, another point that requires discussion is the fact that neuroimaging studies in highly proficient bilinguals have underscored the involvement of subcortical regions during language switching^49^. Here, we used MEG brain recordings, which are more suited to capture cortical activity. Furthermore, our source localization methodological approach is also biased towards detecting cortical regions underlying the sensor-level results. Thus, we cannot completely rule out that some subcortical regions may have also contributed to the observed effects, although our approach was not sensitive enough to detect them.

Third, our study was limited by the absence of a group of low proficient bilinguals. Including individuals with varying levels of language proficiency and diverse linguistic backgrounds in further studies could help capturing bilingualism as a spectrum of experiences that can differentially influence oscillatory brain dynamics. Therefore, future research should aim to address individual variability in L2 proficiency to provide a more comprehensive understanding of bilingual language control.

Finally, contrary to our expectations, no differences were observed in the theta frequency band (4–7 Hz). Theta power increases have been widely implicated in switch cost effects across language switching^30,48,65^ and task switching paradigms^66–69^. For instance, studies showing theta modulations during comprehension^48^ and, particularly, during speech production^30^, report power increases for switches into the L2 as compared to the L1. However, in the studies mentioned above, participants were low-proficiency bilinguals, and thus the executive control demands posed by cross-language interference may have been higher compared to bilinguals with more balanced proficiency levels in their L1 and L2. In line with this interpretation, it has been suggested that theta power increases may reflect higher executive control levels, possibly due to active monitoring of language selection errors^31^ or more demanding lexical search^66^. Thus, the absence of theta effects in our study likely reflects that mechanisms involved in monitoring potential speech errors and/or lexical access became tuned as proficiency increased. Future studies are required in order to disentangle this critical aspect.

## Conclusions

The symmetrical switch and neural costs found in our study suggest that, during speech production, bilinguals with similar proficiency in their L1 and L2 recruit a common, language-independent control mechanism. This mechanism is supported by alpha oscillations sourced in parieto-prefrontal and ATL regions, which mediate language selection and facilitate conceptually-driven lexical access of the intended word.

## Methods

### Participants

Twenty-one Spanish-Basque bilingual speakers (M = 25.04 years; SD = 3.94, 16 females) were recruited through the BCBL *Participa* website (https://www.bcbl.eu/participa/) and received monetary compensation for their participation in the MEG study. Due to technical problems during behavioral data acquisition in the MEG experiment, the onset of verbal reaction times were lost for most of the participants. To compensate for this technical issue, we conducted the same experiment online in an independent sample of twenty-five Spanish-Basque bilinguals (M = 27.4 years; SD = 5.22, 9 females). This latter experiment was conducted online due to the restrictions imposed by the COVID-19 pandemic. Thus, it is worth noting that behavioral and MEG responses come from two independent samples of highly proficient Spanish-Basque bilinguals.

The sample size required for our 2 × 2 factorial design Language (Spanish and Basque) × Trial type (switch and non-switch) was determined with the MorePower software^67^. The expected effect size was set based on previous literature on behavioral switching in highly proficient bilinguals^5–9^ showing that reliable effects can be obtained using partial eta-squared (ηp2) values ranging between 0.25 and 0.5. We used an effect size of ηp2 = 0.35, an alpha level of 0.05 and a desired power of (1 − β) 85%, thus requiring at least 20 participants per group to yield adequate statistical power.

All participants were right-handed as assessed via the Edinburgh Handedness Inventory^68^ and had a normal or corrected-to-normal vision. None of the participants reported major medical, neurological, or psychiatric disorders. Prior to their inclusion in the study, all participants provided their written informed consent. The study protocol was approved by the Ethics Committee of the Basque Center for Cognition, Brain, and Language (BCBL) and was carried out following the Code of Ethics of the World Medical Association (Declaration of Helsinki) for experiments involving humans.

Before the experiments, all participants performed a language background questionnaire to collect detailed information about language age of acquisition and proficiency levels. Language proficiency was measured employing the Basque, English, and Spanish Test [BEST]^69^, using the semi-structured interview part of the test, which taps into fluency, lexical resources, grammatical aspects, and pronunciation (Likert-like scale with scores ranging from 1 to 5). The cut-off criteria for considering an individual as a highly proficient bilingual were scores ≥ 4 in their L2 (and L1). Furthermore, we used a composite score for assessing the percentage of language exposure in Spanish and Basque. The composite scores were calculated by averaging self-reported percentages of listening, writing, and speaking in each language mentioned above. These scores were further normalized using the min–max method. This method is commonly used for data normalization, and it preserves the relationships among the original data values. Higher scores indicate higher exposure to a language and are calculated as follows:

$$v\_i=({v}^{^{\prime}}\_i-min\_A)/(max\_A-min\_A ),$$

where v′ is a new, normalized value, v is the original value, min_A is the minimum value in the range, and max_A is the maximum one.

Overall, participants acquired their two languages in early childhood and had similar proficiency levels and exposure (see Table 1 for detailed information about group profiles).

**Table 1:** Participant’s demographic data and linguistic profiles.

(1) Behavioral experiment		Mean	SD	Contrast between languages		
Age (range: 19–37 years)		27.4	5.22			
Gender		9f. / 16 m				

	Language	Mean	SD	*P*-value	W	rrb
Interview (0–5)	Spanish	4.92	0.28	0.121	68	0.495
	Basque	4.76	0.44			
AoA (0–5 years)	Spanish	0.6	1.7	0.259	29	− 0.363
	Basque	1.32	1.93			
Composite score (0–1)	Spanish	0.38	0.3	0.331	185	0.233
	Basque	0.3	0.24			

(2) MEG experiment		Mean	SD	Contrast between languages		
Age (range: 19–35 years)	25.04	3.94				
Gender		16f./5 m				

	Language	Mean	SD	*P*-value	W	rrb
Interview (0–5)	Spanish	4.96	0.08	0.11	67	0.47
	Basque	4.83	0.29			
AoA (0–4 years)	Spanish	0.47	1.28	0.162	21	− 0.462
	Basque	1.28	1.70			
Composite score (0–1)	Spanish	0.5	0.29	0.10	163	0.41
	Basque	0.3	0.29			

Descriptive statistics and comparisons between languages (performed using paired samples Wilcoxon test) are provided for each group of bilinguals. Mean (M), standard deviations (SD), W values, *p*-values, and effect sizes (rank-biserial correlation coefficients, rrb) are shown for language proficiency indexes (i.e. proxy of language exposure and BEST scores) and age of acquisition (AoA).

Given that most of the participants across experiments (~ 72%) reported Spanish as their dominant language, we will refer to Spanish as L1 and Basque as L2.

### Language switching task

Following previous switching paradigms^5,8^, we selected eight colorful hand-drawn pictures representing high-frequency objects as stimuli [Spanish/Basque names: "Perro"/"Txakurra" (dog); "Ventana"/"Leihoa" (window); "Oso"/"Hartza" (bear); "Gallina"/"Oiloa" (chicken); "Cuchillo"/"Labana" (knife); "Anillo"/"Eraztuna" (ring); "Camisa"/"Alkandora" (shirt); "Oreja"/"Belarria" (ear)]. The pictures were selected from a standardized battery developed by NEURE clinic® (https://www.neure.eu/) and matched in frequency. Frequencies for the Spanish words were calculated using the EsPal database^70^ and those for Basque words were calculated using the E-hirz database^71^. The mean frequency for Spanish words was 22.4 and for Basque 21.8 per million.

**Figure 3:**
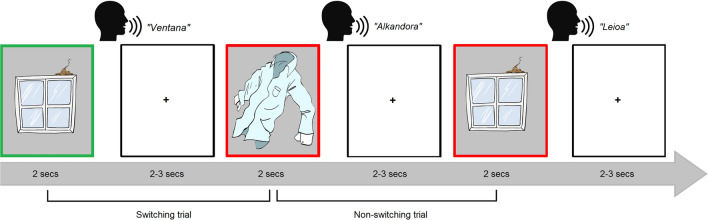
Examples of stimuli and experimental task. Each trial began with a fixation cross on the screen for 2 s followed by the picture presented for 3 s. Participants were requested to overtly name the observed item (e.g. “Camisa”) in either Spanish or Basque depending on a color cue (i.e. picture frame). ISI randomly varied between 2–3 secs.

Participants were asked to overtly name the pictures in either Spanish or Basque depending on a color cue (e.g. red for Spanish and green for Basque) within the same block (i.e. mixed-language context). Participants were asked to name the pictures as fast and accurately as possible. Picture examples and trial structure are depicted in Fig. 3.

A fixation cross appeared on the screen for ~ 2–3 secs. Then the picture was presented for 2 secs, and participants overtly named the observed item (e.g. "Camisa"). The picture remained on the screen for 2 secs regardless of the participants' voice onset. Inter-stimuli interval (ISI) randomly varied between ~ 2–3 secs. Reaction times and accuracy were collected online and stored for further processing offline.

Overall, there were two types of trials: (a) those in which participants had to switch between languages from one trial to another (switch trials), and (b) those in which participants had to name a picture in the same language as in the preceding trial (non-switch trials). Following the seminal work of^2^ in language switching during naming, the proportion of switch to non-switch trials was set to 30/70%. To control for this proportion, the list of the stimuli was pseudo-randomized. More specifically, a total of 336 pseudo-randomized trials were distributed into four conditions: (1) non-switch trials in Spanish (118 trials), (2) non-switch trials in Basque (118 trials), (3) switch trials in Spanish (50 trials), (4) switch trials in Basque (50 trials). Non-switch trials following a switch trial were eliminated to avoid the carry-over effects of switch trials; the first trial was also eliminated as it was impossible to quantify it as a switch or a non-switch trial. After the elimination, on average, 48 trials remained per condition.

The experiment was run at the BCBL facilities while magnetoencephalographic (MEG) signals were simultaneously recorded. The stimuli were presented on a white background in the center of a screen positioned ~ 1 m far from the participant. Participants' verbal responses were recorded using a high-resolution microphone. The online experiment was coded on Javascript using the jsPsych library and was run using the Cognition.run (https://www.cognition.run/) platform. Briefly, participants were instructed to sit in a quiet room to avoid distractions and to have a working microphone on their PCs. In addition, information about the browsers and operating systems required to ensure compatibility and avoid data loss was provided.

### MEG and MRI data acquisition

MEG data were acquired in a magnetically shielded room at a sampling rate of 1000 Hz using a 306-channel (102 magnetometers and 204 planar gradiometers) Elekta Neuromag system (Helsinki, Finland). MEG signals were recorded at a 1 kHz sampling rate and online filtered at a bandwidth of 0.1–330 Hz. Participants' head position inside the helmet was continuously monitored throughout the experiment using 5 head-position indicator (HPI) coils. Six electrode pairs were used to measure horizontal and vertical ocular and cardiac activity. The standard fiducial landmarks (i.e. left and right pre-auricular points and nasion) plus ~ 300 additional points registered over the scalp and eyes/nose contours were digitalized and used to spatially align the MEG sensor coordinates to the native T1 high-resolution 3D structural MRI of each participant. T1s were acquired with a Siemens 3 T magnetom prismafit MR scanner (Siemens, Munich, Germany) in a separate session with the following parameters: echo time = 2.97 ms, non-switching time = 2530 ms, flip angle = 7° and field of view = 256 × 256 × 176 mm3, number of axial slices = 176, slice thickness = 1 mm, in-plane resolution = 1 mm × 1 mm.

### Online behavioral assessment and preprocessing

Verbal responses acquired during the online experiment were recorded using the participant's hardware of choice (e.g. headphones, microphone, built-in microphone). For safety reasons and to make online data collection possible, the audio files were recorded as .webm files encoded in a base64 string. For processing speech data, a semi-automated open-source in-house software ("SPONGE") was developed using Python (https://github.com/Polina418/Audio_processing). The software was used to decode and convert the audio files into .wav format, semi-automatically detect speech onsets (with manual online correction), and perform speech recognition using Google Cloud Speech-to-Text API. Speech recognition results were manually corrected offline.

Reaction times were measured as the interval between picture presentation and the onset of the participant's verbal response, disregarding all background noise preceding the target response. Participants had a maximum of 3 s to give their responses. Trials in which the participant made a mistake or mumbled prior to the target word (e.g. "Hmmm dog") were excluded from further analysis (~ 3%). Four participants were removed from the online experiment due to the low quality of the audio recordings. Thus, the final analysis was performed on a reduced sample of twenty-one participants.

### MEG data acquisition and preprocessing

Continuous data were preprocessed offline using the temporal extension of the signal space separation method (tSSS)^72^ implemented in Maxfilter 2.2 (Elekta-Neuromag). Briefly, tSSS subtracts external magnetic noise from the MEG recordings, corrects for head movements, and interpolates bad channels. Subsequent analyses were performed using the MatlabR2014B and FieldTrip toolbox version 20,170,911^73^. Recordings were down-sampled to 500 Hz and segmented into epochs time-locked to picture presentation from 1000 ms before image onset to 1000 ms after image onset.

A semi-automatic procedure was employed to remove epochs containing electromyographic artifacts, SQUID jumps, and flat signals. Finally, a fast independent component analysis (fast ICA) was used to identify components reflecting blinks and electrocardiographic artifacts^74^. Two participants were discarded from the final analysis due to a high number of blinking/muscular artifacts in the MEG recording (e.g. leading to > 40 kept trials in some conditions). Thus, the final MEG analysis was performed on a reduced sample of nineteen participants.

### Data analysis

#### Behavioral analysis

Naming accuracy in all participants was at ceiling (Spanish Mean = 99.38%, SD = 1.14; Basque Mean = 99.49%, SD = 0.96); thus, statistical analyses were only performed on the RTs. RTs analyses were conducted on the correct trials. Individual RTs were subjected to a 2-way ANOVA with Language (Spanish, Basque) and Trial type (switch, non-switch) as within-subject variables. Furthermore, we paralleled the frequentist null-hypothesis significance testing with a Bayesian ANOVA approach to provide a more comprehensive assessment of the evidence for both the null and alternative hypotheses. This analysis was implemented in the JASP software^41^.

Finally, to properly model by-participant and by-item variation, we paralleled the ANOVA using LMMs analysis. As fixed effects, we entered Language, Trial type, and their interaction into the model. Apart from the fixed effects, the model included Participants and Items as random effects (random intercepts and random slopes). The two-level categorical predictors were coded as − 0.5 and 0.5 (i.e. Basque trials were coded as − 0.5 and Spanish trials as 0.5; non-switch trials were coded as − 0.5 and switch trials as 0.5). *P*-values were obtained using the Satterthwaite's method. The analysis was performed in R (R Core Team, version 4.1.3) using the lme4 package (version 1.1–29)^75^ and lmerTest package (version 3.1–3)^76^.

#### Time–frequency analysis

Time–frequency representations (TFR) were calculated on clean MEG data epochs in the theta (4–7 Hz) and the alpha (8–13 Hz) frequency bands. These frequency bands were selected based on a recent review covering findings regarding neural responses to bilingual language control in diverse tasks^17^. TFRs were obtained using a Hanning tapers approach and a fixed window length of 500 ms, advancing in 10 ms steps. Power was estimated separately for each orthogonal direction of a gradiometer pair and then combined, resulting in 102 measurement sensors. Magnetometers were discarded due to low signal-to-noise ratio. Power was calculated as the relative change with respect to a ~ 500 ms pre-stimulus baseline. We used cluster-based permutation tests for the statistical contrasts at the sensor level^77^. We averaged over frequency bins and time points in two specific time windows of interest to assess power differences: 100-350 ms and 350–600 ms after picture onset. These time windows were chosen based on data inspection and neurophysiological evidence from studies using speech production tasks^39,47,78^, suggesting that recordings not contaminated with articulatory activity can be safely acquired around these time windows.

The permutation *p*-value was calculated using the Monte-Carlo method with 1000 random permutations. The significance testing threshold was a *p*-value below 5% (two-tailed).

#### Source reconstruction

Source reconstruction was performed on the statistically significant effects observed at the sensor level. For each participant, individual T1-weighted MRI images were segmented into different brain tissues using the Freesurfer software. Co-registration between the MEG sensor space and participant's MRI coordinates was done by manually aligning the digitized head-surface and fiducial points to the outer scalp surface using MRIlab (Elekta Neuromag Oy, version 1.7.25). The forward model was calculated using the Boundary Element Method (BEM) implemented in the MNE suite (RRID: SCR_005972)^79^ for three orthogonal tangential current dipoles, placed on a homogeneous 5-mm grid covering the whole brain. The forward model was reduced to the two principal components of the highest singular value for each source, corresponding to sources tangential to the skull. All sensors (i.e. planar gradiometers and magnetometers) were used for source estimation, normalizing the signal of each sensor by its noise variance (i.e. 500 ms baseline period before picture onset). Brain source activity was calculated using a Linearly Constrained Minimum Variance (LCMV) beamformer approach^80^. The covariance matrix used to derive beamformer weights was estimated from the time–frequency window of the significant sensor-level effects and an equally-sized baseline period prior to picture onset. In order to perform group-level analysis, brain maps were transformed from the individual MRIs to the standard Montreal Neurological Institute (MNI) by applying a non-linear transformation using the spatial-normalization algorithm implemented in SPM8.

Comparisons between conditions were performed with the location-comparison method^81^. This method generates bootstrap group-averaged maps to build a permutation distribution of location difference between local maxima in the two conditions being compared and test the null hypothesis that this distance is zero. Local maxima are defined as sets of contiguous voxels displaying higher power than all other neighboring voxels. The threshold for statistical testing at *p* < 0.05 was computed as the 95-percentile of the permutation distribution. All supra-threshold local MEG peaks were interpreted as indicative of brain regions likely triggering the sensor-level effects. The coordinates of significant local power maxima were statistically compared across participants using *t* tests.

## Data Availability

All the data that support the findings of this study are available at https://osf.io/td7xn/. The code for data preprocessing and analysis are available on request from the corresponding author.
